# Utilization of primary and secondary biochemical compounds in cotton as diagnostic markers for measuring resistance to cotton leaf curl virus

**DOI:** 10.3389/fpls.2023.1185337

**Published:** 2023-06-06

**Authors:** Prashant Chauhan, Naresh Mehta, R. S. Chauhan, Abhishek Kumar, Harbinder Singh, Milan Kumar Lal, Rahul Kumar Tiwari, Ravinder Kumar

**Affiliations:** ^1^ College of Agriculture, Chaudhary Charan Singh (CCS) Haryana Agricultural University, Hisar, India; ^2^ Department of Plant Pathology, Chaudhary Charan Singh (CCS) Haryana Agricultural University, Hisar, India; ^3^ Krishi Vigyan Kendra, Chaudhary Charan Singh (CCS) Haryana Agricultural University, Hisar, India; ^4^ Department of Crop Physiology, Biochemistry & Postharvest Technology, Indian Council of Agricultural Research (ICAR)-Central Potato Research Institute, Shimla, Himachal Pradesh, India; ^5^ Department of Plant Protection, Indian Council of Agricultural Research (ICAR)-Central Potato Research Institute, Himachal Pradesh, India

**Keywords:** cotton, cotton leaf curl virus, biochemical, CLCuD, resistance

## Abstract

**Introduction:**

Cotton (*Gossypium hirsutum* L.) is one of the most important staple fibrous crops cultivated in India and globally. However, its production and quality are greatly hampered by cotton leaf curl disease (CLCuD) caused by cotton leaf curl virus (CLCuV). Therefore, the aim of the present study was to investigate the biochemical mechanisms associated with CLCuD resistance in contrasting cotton genotypes.

**Methods:**

Four commercial cotton varieties with susceptible (HS 6 and RCH-134 BG-II) and resistant (HS 1236 and Bunty) responses were used to analyze the role of primary (sugar, protein, and chlorophyll) and secondary (gossypol, phenol, and tannin) biochemical compounds produced by the plants against infection by CLCuV. The resistant cultivars with increased activity of protein, phenol, and tannin exhibited biochemical barriers against CLCuV infection, imparting resistance in cotton cultivars.

**Results:**

Reducing sugar in the healthy plants of the susceptible Bt cultivar RCH 134 BG-II exhibited the highest value of 1.67 mg/g at 90 days. In contrast, the lowest value of 0.07 mg g^-1^ was observed at 60 DAS in the highly diseased plants of the susceptible hybrid HS 6. Higher phenol content (0.70 mg g^-1^) was observed at 90 DAS in resistant cultivars, whereas highly susceptible plants exhibited the least phenol (0.25 mg g^-1^) at 90 DAS. The lowest protein activity was observed at 120 DAS in susceptible cultivars HS 6 (9.4 mg g^-1^) followed by RCH 134 BG-II (10.5 mg g^-1^). However, other biochemical compounds, including chlorophyll, sugar, and gossypol, did not show a significant role in resistance against CLCuV. The disease progression analysis in susceptible cultivars revealed non-significant differences between the two susceptible varieties.

**Discussion:**

Nevertheless, these compounds are virtually associated with the basic physiological and metabolic mechanisms of cotton plants. Among the primary biochemical compounds, only protein activity was proposed as the first line of defense in cotton against CLCuV. The secondary level of defense line in resistance showed the activity of secondary biochemical compounds phenol and tannins, which displayed a significant increase in their levels while imparting resistance against CLCuV in cotton.

## 1 Introduction

Cotton (*Gossypium hirsutum*) is a staple fibrous crop cultivated in the sub-tropical and seasonally dry regions of the northern and southern hemispheres. India is the largest producer of cotton on the global map, contributing to 23% of its production (Barwale, 2016; Anonymous, 2019). However, global cotton production has shown a decline of approximately 2% in 2018 due to biotic and abiotic stresses. Nevertheless, an overall 6% yield growth of cotton can help achieve the global demand for cotton by 2028 (Anonymous, 2019). Cotton leaf curl disease (CLCuD) caused by cotton leaf curl virus (CLCuV) is one of the most devastating and prominent diseases of cotton in the Indian subcontinent (Farooq et al., 2011; Monga, 2014; Qadir et al., 2019). The virus is a begomivirus of the family Geminiviridae and is closely associated with satellite molecules (beta satellite and alpha satellite). It is transmitted by whitefly (*Bemisiatabaci* Gem.) through circulative persistent transmission in plants (Sharma and Rishi, 2003; Kumar et al., 2021). CLCuV poses a significant threat to cotton production around the world, causing substantial yield loss and economic harm. The CLCuV is transmitted by whiteflies and causes crinkled, misshapen, and discolored leaves in infected cotton plants, severely reducing their growth and output. This virus poses a significant challenge in cotton-growing regions, especially in major cotton-producing nations like Pakistan, India, and China. Widespread crop damage caused by CLCuV has resulted in significant economic losses for farmers and the cotton sector (Farooq et al., 2011; Monga, 2014; Qadir et al., 2019). To combat the problem, various techniques have been developed, such as adopting virus-resistant cotton types, implementing integrated pest management practices to control whitefly populations, and creating fast diagnostic tools to detect the virus early. Despite these efforts, CLCuV remains a chronic concern, and its control is still a priority in global cotton production.

The acquisition of the virus by whiteflies may vary due to biochemical and genetic differences in whitefly races with their host (Yang et al., 2011). Additionally, the compositional differences in host saps could affect the behavior and growth of the insects (Gupta et al., 2010). Understanding the biochemical responses of different varieties can enhance the management of CLCuD and its vector, the whitefly. Plants rely heavily on biochemical compounds to defend against numerous biotic stress factors, such as diseases and insect pests (Iqbal et al., 2021). Sugars and phenols provide the plant with energy and also act as signaling molecules to activate defense responses (Lal et al., 2022; Kumar et al., 2023). In response to pathogen attacks, plants produce secondary metabolites such as antifungal and antibacterial phytoalexins. Pathogenesis-related (PR) proteins and other proteins are involved in cell defense, cell wall reinforcement, and acquired systemic resistance. Insects feed on chlorophyll because it is necessary for photosynthesis and a food source for plants. The degradation of chlorophyll can produce harmful by-products, which can discourage feeding. Phenols are essential components of lignin and suberin, which reinforce plant cell walls and inhibit disease penetration. Gossypol, which is present in cotton plants, is poisonous to numerous insect pests and functions as a defense mechanism. Tannins are astringent secondary metabolites with antifungal and antibacterial effects that can discourage insect feeding.

Biochemical compounds play a crucial role in the survival and reproduction of plants under biotic stress (Wilson and Smith, 1976; Hedin and McCarty, 1990; Borkar and Verma, 1991; Bhat, 1997; Chakrabarty et al., 2002; Singh and Agarwal, 2004; Beniwal et al., 2006; Acharya and Singh, 2008; Govindappa et al., 2008; Ajmal et al., 2011; Anuradha, 2014). Although their association with plant response to biotic stresses has been extensively studied, little research has been conducted on the response of plants to virus-induced diseases (Kumar et al., 2020; Kumar et al., 2021; Kumar et al., 2022). To address this gap, we investigated the biochemical response of cotton plants to CLCuD by examining the production of various primary and secondary metabolites. This study can provide valuable insights into the mechanism involved in the resistance against CLCuV.

## 2 Materials and methods

### 2.1 Plant genotypes and analytical parameters

Four commercially grown cotton cultivars were selected for the study based on their disease incidence and plant types. Two hybrids, HS6 and H 1236, and two genetically transformed *Bacillus thuringiensis* (Bt) varieties with Bt genes against bollworms (RCH-134 BG-II and Bunty) were chosen for the present study. Based on the previous studies on response against CLCuV, the cultivars H 1236 and Bunty were considered resistant, whereas HS 6 and RCH-134 BG-II were the susceptible sources in the present study (Anonymous, 2018). The experiment was conducted during the summer (*Kharif*) season using a randomized block design in plots measuring 5 × 4 m^2^ in four replications. The disease appeared in 1-month-old plants along with an observed buildup of white fly populations. The disease was allowed to spread under natural epiphytic conditions (**Supplementary Figures 1A, B**). Standard biochemical methods were used to analyze the levels of sugar (total and reducing sugar), proteins, chlorophyll-a and chlorophyll-b, gossypol, total phenols, and tannins. Plants were scored on a disease grade scale ranging from 1 to 6, with grades 1–2 indicating a resistant (R) response, grade 3 indicating a moderately resistant (MR) response, grade 4 indicating a moderately susceptible (MS) response, and grades 5–6 indicating a susceptible (S) response to CLCuD (**Table 1**). Representative leaf samples of the CLCuD diseased plants of the four cotton varieties raised in the field conditions were collected. Plants with disease grades 0, 2, 4, and 6 of all four cultivars were selected at 40, 60, 90, and 120 days after sowing to observe the response of plants to disease infection at different growth stages.

**Table 1:** Disease scale used for grading of CLCuD.

Grade of disease	Reaction response	Plant response
1	R	Thickening of small veins
2		Grade 1 + main vein thickening and little leaf curling
3	MR	From top 1/4 of the plant showing leaf curling
4	MS	Upper 1/2 of the plant affected by leaf curling
5	S	From top 3/4 of the plant affected by leaf curling
6		Severe stunting of plant with leaf curling

### 2.2 Leaf sample preparation

Symptomless leaf samples were collected from resistant cultivars, while leaves of different disease grades (2, 4, and 6) were used from susceptible cultivars for analysis. In order to ensure a fair comparison, leaves without symptoms from susceptible cultivars were used as controls from the experimental plots under natural epiphytic exposure to white fly (vector of CLCuV) and the virus. The collected leaves were cleaned with sterilized water to remove any foreign material from the surface and sun-dried for 3 days. The dried samples were subjected to oven drying at 60°C for 5 days until completely dried. The leaves were then crushed into a fine powder for biochemical analysis, whereas fresh leaf samples were taken for chlorophyll estimation. The plants of susceptible cultivars exhibited a higher disease index of grade 4 after 30 days of sowing; therefore, biochemical response for various parameters for highly diseased plants was performed during the second stage of sampling.

### 2.3 Quantitative determination of primary metabolites

#### 2.3.1 Sugar determination

Each test tube containing 100 mg of powdered leaf samples was added with 5 ml of 80% ethanol. These tubes were then placed in a hot water bath for 25–30 min at 80°C and mixed well. After cooling to room temperature, the tubes were centrifuged at 4,000 rpm for 10 min. The resulting supernatant was transferred to another set of test tubes for each sample and made up to a final volume of 10 ml with distilled water. The sugar content was estimated using the phenol-sulfuric acid method (DuBois et al., 1956).

#### 2.3.2 Protein determination

The 100 mg of leaf extract was taken and poured into a 150-ml digestion flask. A 10-ml solution of H_2_SO_4_ and HClO_4_ in the ratio of 4:1 was then gently poured along the walls of the flask. The mixture was left undisturbed for 24 h. Afterward, the flask was heated on a hot plate until the solution became colorless and was then allowed to cool at room temperature. The final volume was made 100 ml by adding distilled water and was transferred to the distillation apparatus. The total protein content was analyzed from the leaves using the Kjeldahl method as described by AOAC (Helrich, 1990).

#### 2.3.3 Chlorophyll-a and chlorophyll-b determination

The determination of chlorophyll-a and b was carried out following the method of Hiscox and Israelstam (1979), and the formulas proposed by Arnon (1949) were used to calculate the total chlorophyll-a and chlorophyll-b as follows:

Total chlorophyll (mg g^−1^)


$$=\;\frac{(20.2\;x\;A_{645})+(8.02\;x\;A_{663})\;\times \;V}{\left(1,000\;\times \;W\right)}$$

Chlorophyll-a (mg g^−1^)


$$=\;\frac{(12.7\;x\;A_{663})\;x\;(2.69\;x\;A_{645})\;\times \;V}{\left(1,000\;\times \;W\right)}$$

Chlorophyll-b (mg g^−1^)


$$=\;\frac{(22.9\;x\;A_{645})+\;(4.69\;x\;A_{663})\;\times \;V}{1,000\;\times \;W}$$

where *A*_663_ and *A*_645_ = absorbance at wavelengths 645 and 663 nm, respectively;

*V* = volume of solution; and

*W* = weight of sample.

### 2.4 Quantitative determination of secondary metabolites

#### 2.4.1 Gossypol determination

For each sample, 500 mg of powdered cotton leaves was taken and added to a 25-ml conical flask. Then, 10 ml of ethyl alcohol (95%) was added to the flask. The samples were subjected to a hot water bath for 5 min, filtered into fresh test tubes, and centrifuged at 8,000 rpm for 15 min at 18°C. Dilution was performed by adding 40% ethanol, and 1 N HCl was used to adjust the pH to 3.0. Using a separating funnel, 1.5 ml of diethyl ether at 10°C was mixed into the content of the test tubes. The extract was allowed to evaporate until the tubes were dried, and gossypol was estimated by using Diels–Alder reaction of hemigossypolone with myreene as per the method described by Bell (1986).

#### 2.4.2 Phenol determination

Up to extract preparation for phenol determination, the method was the same as that used for sugar determination (*Section 2.3.1*). The phenol content was estimated using Folin-Ciocalteu’s agent and the standard method described by Bray and Thorpe (1954).

#### 2.4.3 Tannin determination

For each leaf extract, 100 mg was taken and added to a 10-ml oak ridge tube along with 5 ml of 70% acetone. The tubes were subjected to a hot water bath at 70°C for 25–30 min and then vortexed to ensure thorough mixing. After cooling to room temperature, the tubes were centrifuged at 4,000 rpm for 10 min. The resulting supernatant was transferred to fresh, empty oak ridge tubes for tannin estimation, following the method described by Porter et al. (1986).

### 2.5 Disease appearance, percent incidence, and progression

The disease appearance, incidence, and progression were recorded for 50 selected plants in the field for two susceptible cultivars grown in the same field. Weekly observations on disease development showed that the disease first appeared on June 21 (3rd week of crop stage) in both cultivars, and reached a maximum (100%) in the 9th week of the crop stage after infection (**Table 2**). In the second-year trial, the disease appeared on June 30 (5th week of crop stage) and reached its maximum (100%) during the 10th week of the crop stage (**Table 3**). The varietal behavior of CLCuD was also recorded, revealing that both cultivars were susceptible to CLCuD, and there were no variations in the development of CLCuD.

**Table 2:** CLCuD disease incidence and periodical disease progression on two cotton cultivars.

Crop age	HS-6*			RCH 134 BG-II**		
Days after sowing	DS (%)	Disease incidence (%)	Disease progression (%)	DS (%)	Disease incidence (%)	Disease progression (%)
**23**	1.03	1.5	1.5	1.30	1.2	1.2
**30**	6.30	17.3	15.8	8.37	13.3	12.1
**37**	9.01	55.7	38.4	11.58	24.0	10.7
**44**	12.66	86.7	31.0	16.02	84.3	60.3
**51**	18.25	97.6	10.9	20.11	95.3	11.0
**58**	24.76	100.0	2.4	26.30	100.0	4.7
**65**	32.03	100.0	0.0	38.45	100.0	0.0
**72**	38.62	100.0	0.0	46.60	100.0	0.0
**79**	43.26	100.0	0.0	51.13	100.0	0.0
**86**	47.74	100.0	0.0	56.10	100.0	0.0
**93**	52.92	100.0	0.0	73.28	100.0	0.0
**100**	57.64	100.0	0.0	66.04	100.0	0.0
**107**	62.33	100.0	0.0	70.29	100.0	0.0
**114**	68.76	100.0	0.0	76.84	100.0	0.0
**121**	72.55	100.0	0.0	77.05	100.0	0.0

*Hybrid cotton, **Bt cotton.

**Table 3:** CLCuD disease incidence and periodical disease progression on two cotton cultivars during 2014.

Crop age	HS-6*			RCH 134 BG-II**		
Days after sowing	DS (%)	Disease incidence (%)	Disease progression (%)	DS (%)	Disease incidence (%)	Disease progression (%)
**30**	1.10	6.8	6.8	2.00	12.3	12.3
**37**	2.00	22.5	15.7	6.40	27.8	15.5
**44**	7.50	49.3	26.8	13.00	59.3	31.5
**51**	12.18	82.3	33.0	16.55	93.7	34.4
**58**	17.83	95.7	13.4	21.28	98.4	4.7
**65**	24.70	100.0	4.3	24.90	100.0	1.6
**72**	29.80	100.0	0.0	32.80	100.0	0.0
**79**	33.00	100.0	0.0	37.10	100.0	0.0
**86**	36.10	100.0	0.0	40.40	100.0	0.0
**93**	38.60	100.0	0.0	43.70	100.0	0.0
**100**	40.90	100.0	0.0	48.00	100.0	0.0
**107**	43.70	100.0	0.0	50.70	100.0	0.0
**114**	50.00	100.0	0.0	53.74	100.0	0.0
**121**	57.10	100.0	0.0	57.30	100.0	0.0

*Hybrid cotton, **Bt cotton.

### 2.6 Statistical analysis

The data obtained were analyzed using the online statistical tool OPSTAT (hau.ernet.in/about/opstat.php). Two-way ANOVA was performed to analyze the observations for both years. This analysis allowed for a comprehensive understanding of the data and enabled the identification of any significant differences or relationships between the variables studied.

## 3 Results

### 3.1 Sugar (total and reducing)

**Figure 1:**
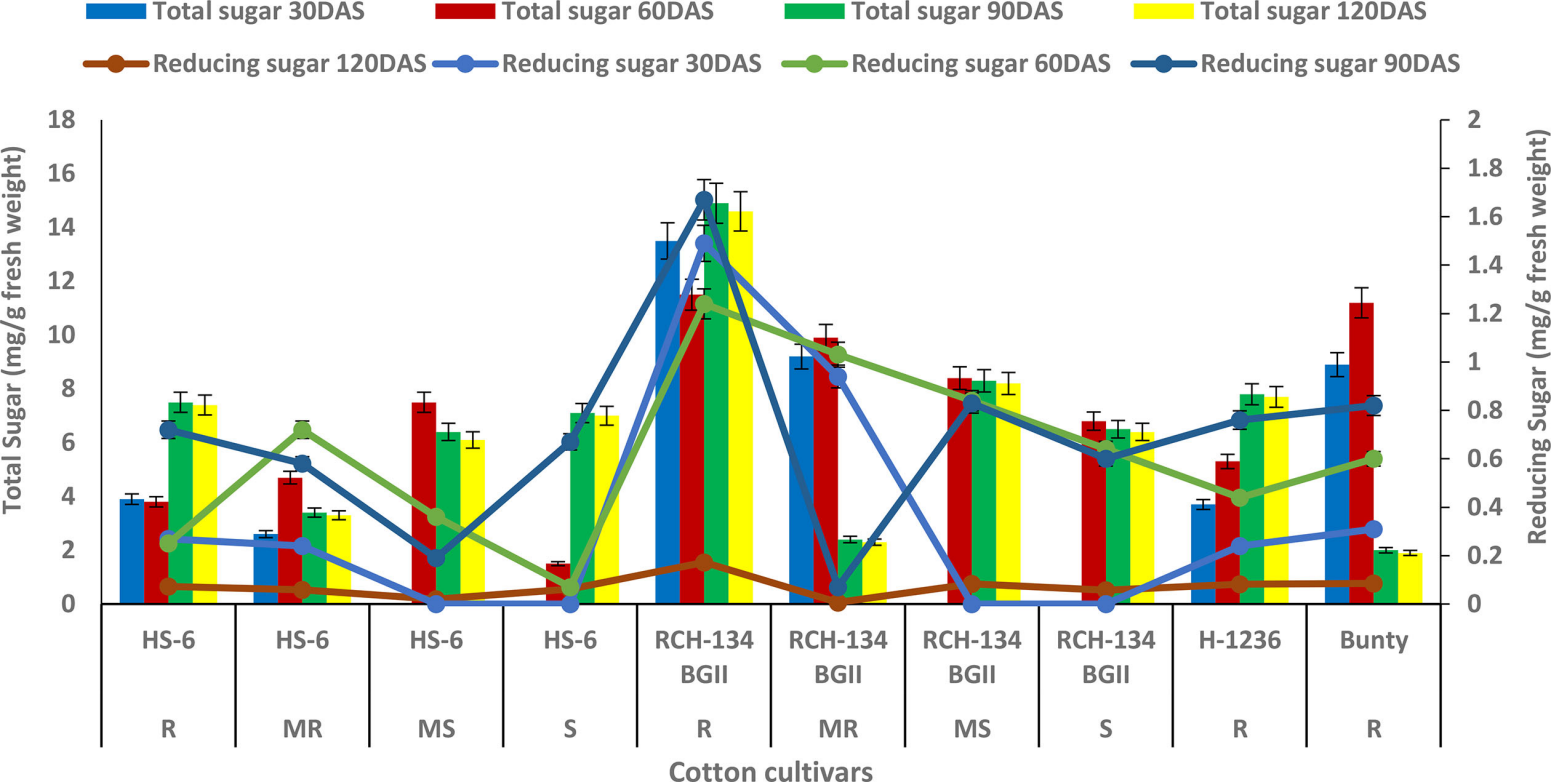
Total sugar (bars) and reducing sugar (lines) in cotton cultivars estimated at 30, 60, 90, and 120 days after sowing. The susceptible cultivars were Non-Bt (HS 6) and Bt (RCH 134 BGII). Non-Bt H1236 and Bt Bunty were taken as resistant cotton cultivars.

The maximum total sugar content of 14.9 mg g^−1^ was observed in symptomless plants of the susceptible cultivar RCH 134 BG-II at 90 DAS (as shown in **Figure 1**) under natural epiphytotic conditions. In contrast, the lowest sugar content of 1.5 mg g^−1^ was found in highly diseased plants of the susceptible hybrid HS 6 at 60 DAS. The resistant cultivars, Bunty, exhibited a decrease in total sugar at 120 DAS. Additionally, the reducing sugar in healthy plants of the susceptible Bt cultivar RCH 134 BG-II was the highest at 1.67 mg/g at 90 DAS (**Figure 1**). Conversely, the lowest value of 0.07 mg g^−1^ was observed at 60 DAS in highly diseased plants of the susceptible hybrid HS 6.

### 3.2 Protein

**Figure 2:**
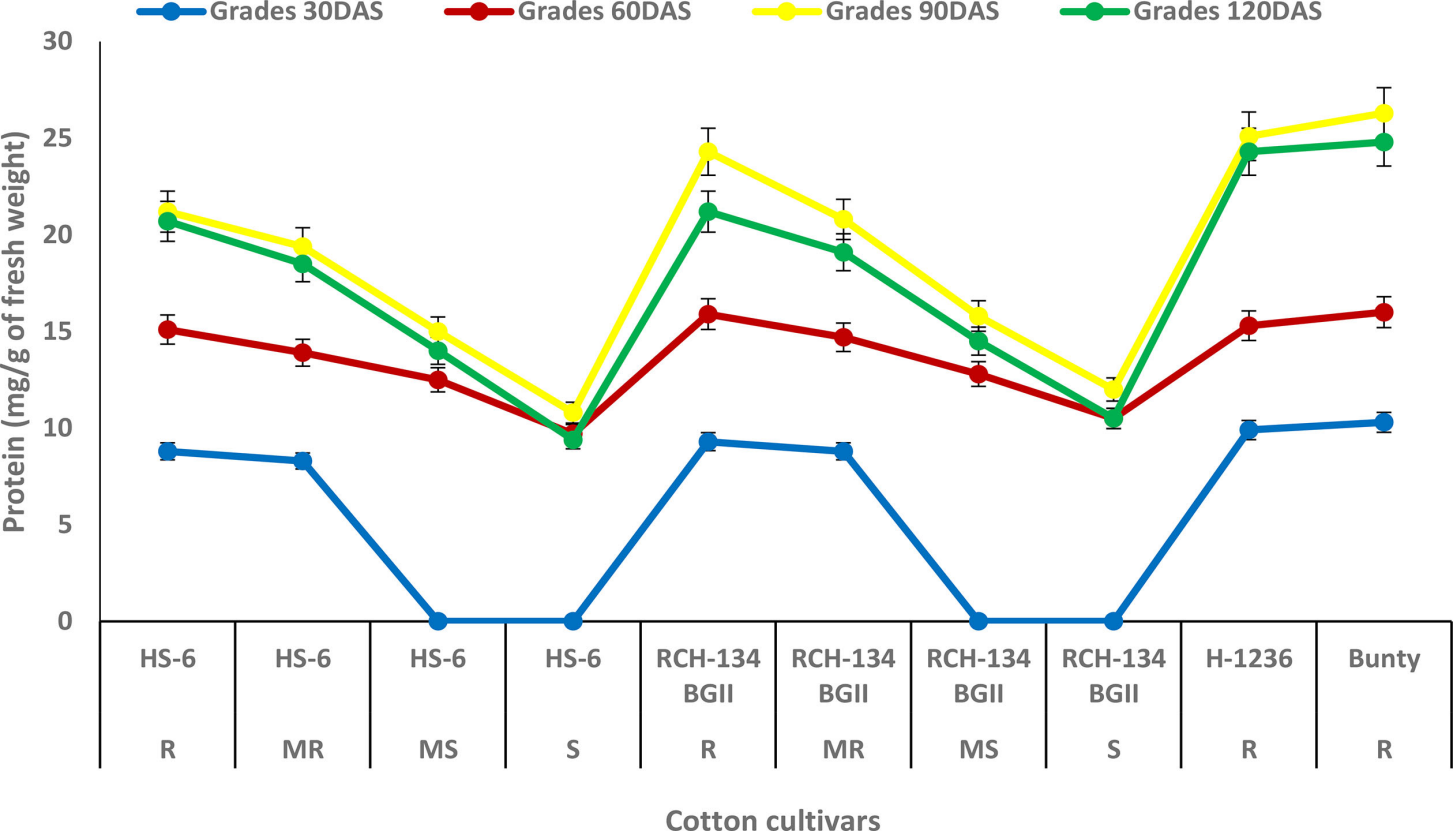
Protein content in cotton cultivars estimated at 30, 60, 90, and 120 days after sowing. The susceptible cultivars were Non-Bt (HS 6) and Bt (RCH 134 BGII). Non-Bt H1236 and Bt Bunty were taken as resistant cotton cultivars.

The maximum protein content of 26.3 mg g^−1^ was observed at 90 DAS in Bunty, followed by the resistant hybrid H 1236 with 25.1 mg g^−1^ (**Figure 2**). Even the healthy plants of the susceptible cultivars exhibited an increase in protein content. The susceptible cultivars HS 6 and RCH 134 BG-II, with lower disease incidence, exhibited more protein as compared to highly diseased plants. The lowest protein content was observed at 120 DAS in the susceptible cultivars HS 6 (9.4 mg g^−1^) followed by RCH 134 BG-II (10.5 mg g^−1^).

### 3.3 Chlorophyll (a and b)

**Figure 3:**
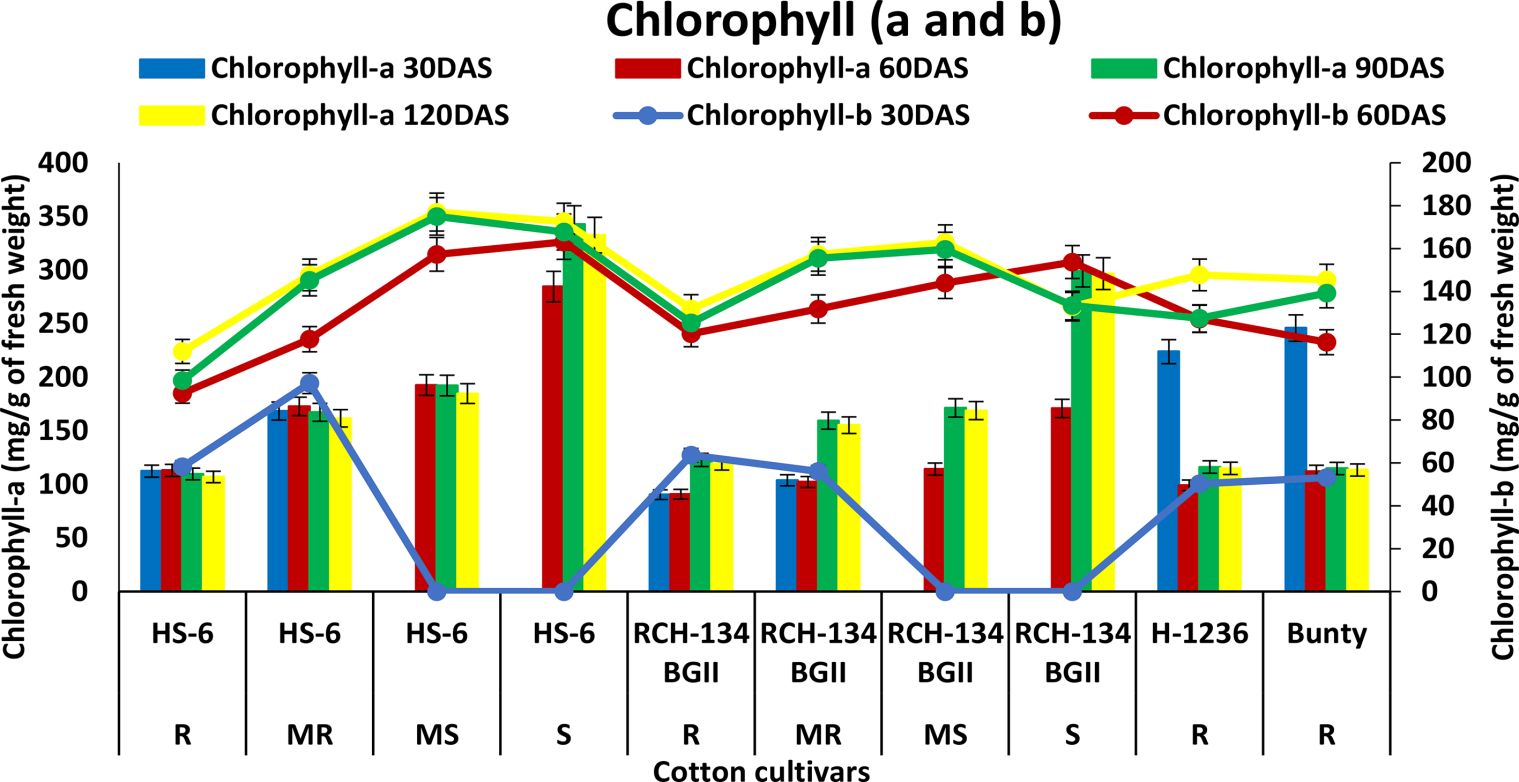
Chlorophyll-a (bars) and chlorophyll-b (lines) content in cotton cultivars estimated at 30, 60, 90, and 120 days after sowing. The susceptible cultivars were Non-Bt (HS 6) and Bt (RCH 134 BGII) with varied disease reactions. Non-Bt H1236 and Bt Bunty were taken as resistant cotton cultivars.

An increase in chlorophyll-a (Chl-a) was observed with the progression of CLCuD symptoms and crop growth stages. HS 6 and RCH 134 BG-II exhibited increased Chl-a levels up to 90 DAS (**Figure 3**). Highly diseased plants of susceptible cultivars HS 6 (342.8 mg g^−1^) and RCH 134 BG-II (299.1 mg g^−1^) exhibited higher Chl-a levels at 90 DAS. In contrast, resistant cultivars H 1236 and Bunty exhibited 223.8 mg g^−1^ and 245.8 mg g^−1^ Chl-a, respectively, at 30 DAS. Chlorophyll-b (Chl-b) levels remained low in all cultivars up to 30 DAS.

### 3.4 Phenol

**Figure 4:**
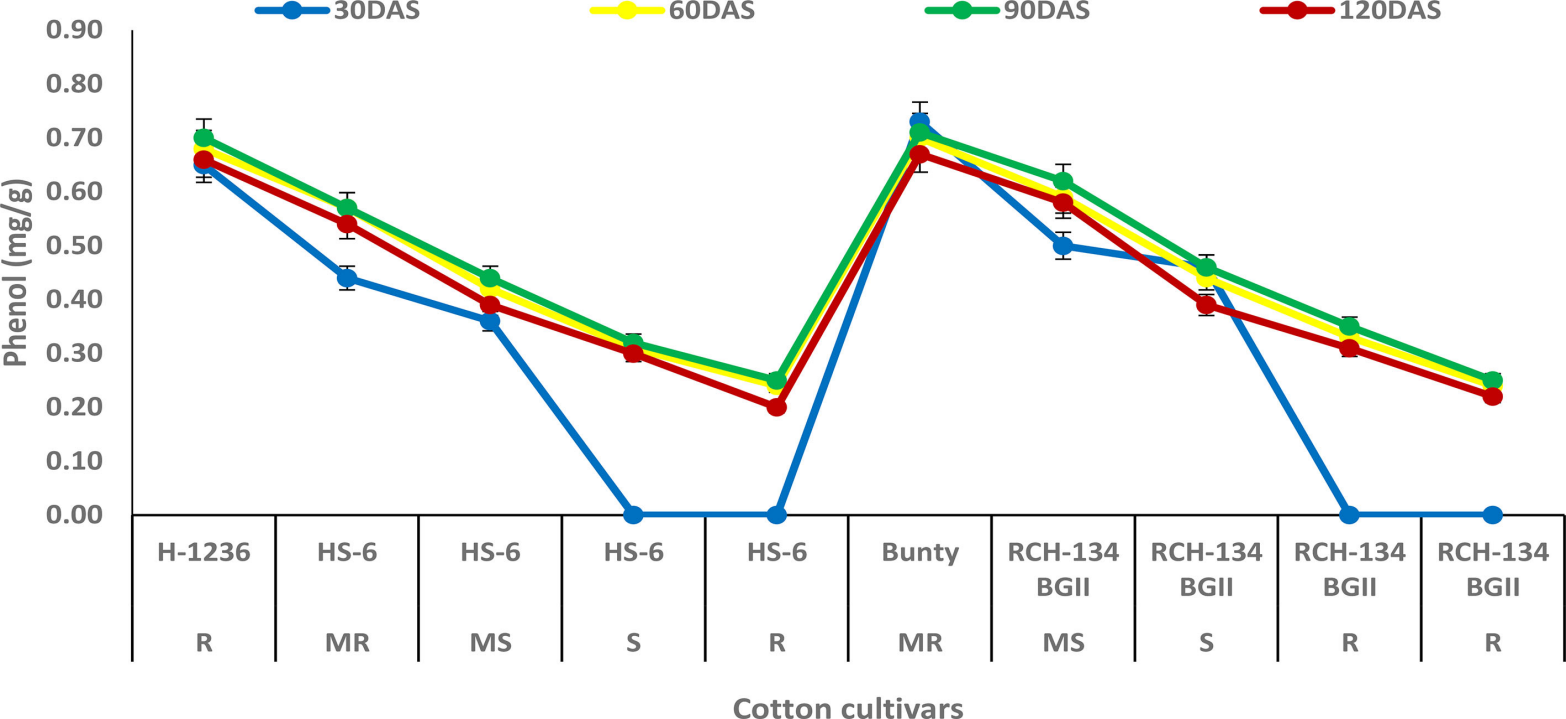
Phenol content in cotton cultivars estimated at 30, 60, 90, and 120 days after sowing. The susceptible cultivars were Non-Bt (HS 6) and Bt (RCH 134 BGII) with varied disease reactions. H1236 (Non-Bt) and Bunty (Bt) were taken as resistant cultivars.

The phenol content was observed to increase with plant growth up to 90 DAS, but decreased at 120 DAS (**Figure 4**). At 90 DAS, higher phenol content (0.70 mg g^−1^) was observed in Bunty and H 1236, while highly diseased plants of HS 6 and RCH 134 BG-II exhibited the least phenol content (0.25 mg g^−1^). Notably, highly diseased plants in susceptible cultivars showed lower phenol content compared to the resistant cultivars.

### 3.5 Gossypol

**Figure 5:**
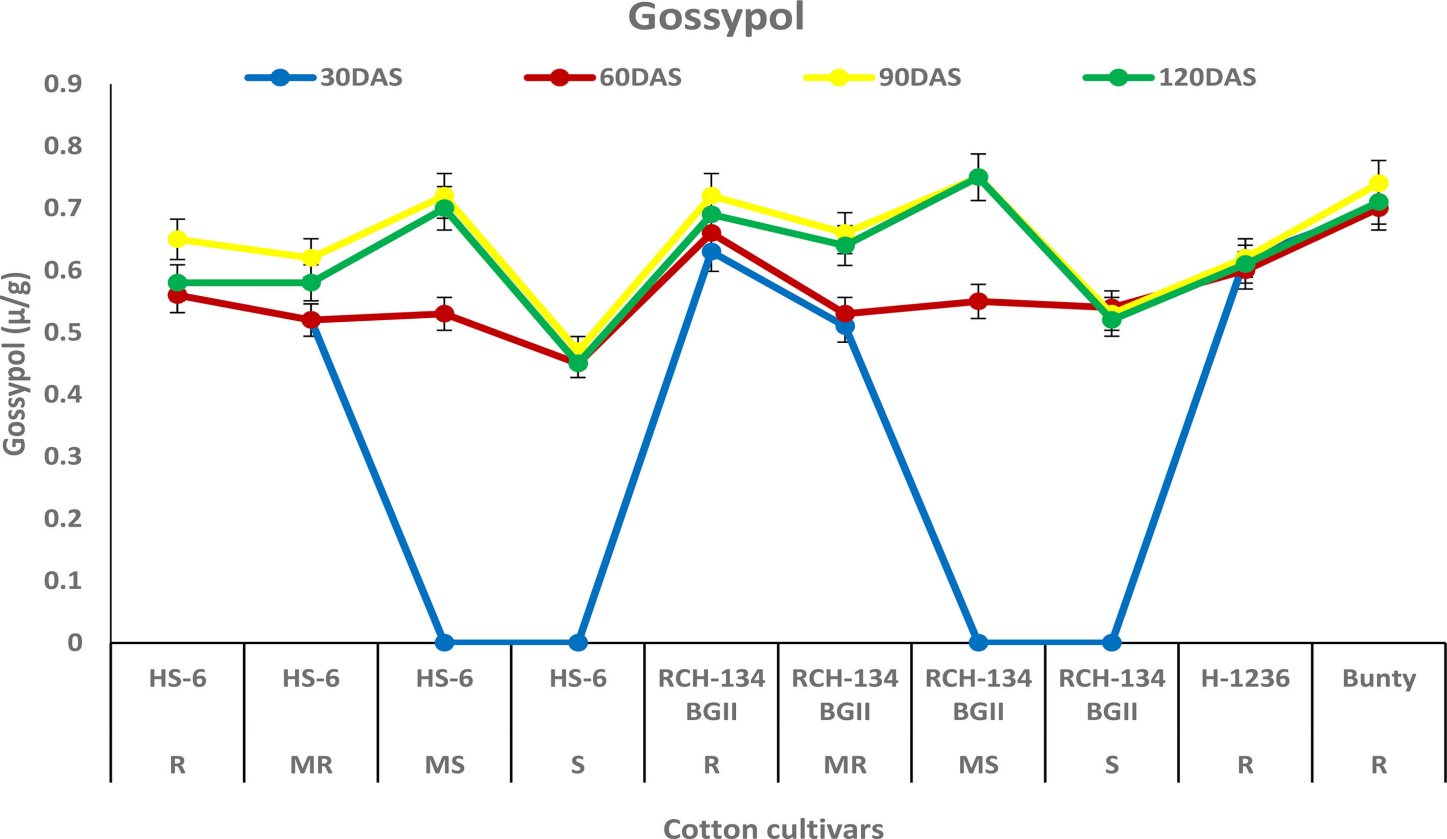
Gossypol in cotton cultivars estimated at 30, 60, 90, and 120 days after sowing. The susceptible cultivars were Non-Bt (HS 6) and Bt (RCH 134 BGII) with varied disease reactions. H1236 (Non-Bt) and Bunty (Bt) were taken as resistant cultivars.

The concentration of gossypol increased in all four cultivars up to 90 DAS (**Figure 5**). The susceptible cultivar RCH 134 BG-II and the resistant cultivar Bunty exhibited the highest gossypol content, measuring 0.75 µg/g and 0.74 µg/g, respectively. At 90 DAS, the symptomless susceptible cultivars RCH 134 BG-II and HS 6 had the same gossypol content, measuring 0.72 µg/g. On the other hand, highly diseased plants of the same cultivars exhibited the lowest gossypol content.

### 3.6 Tannin

**Figure 6:**
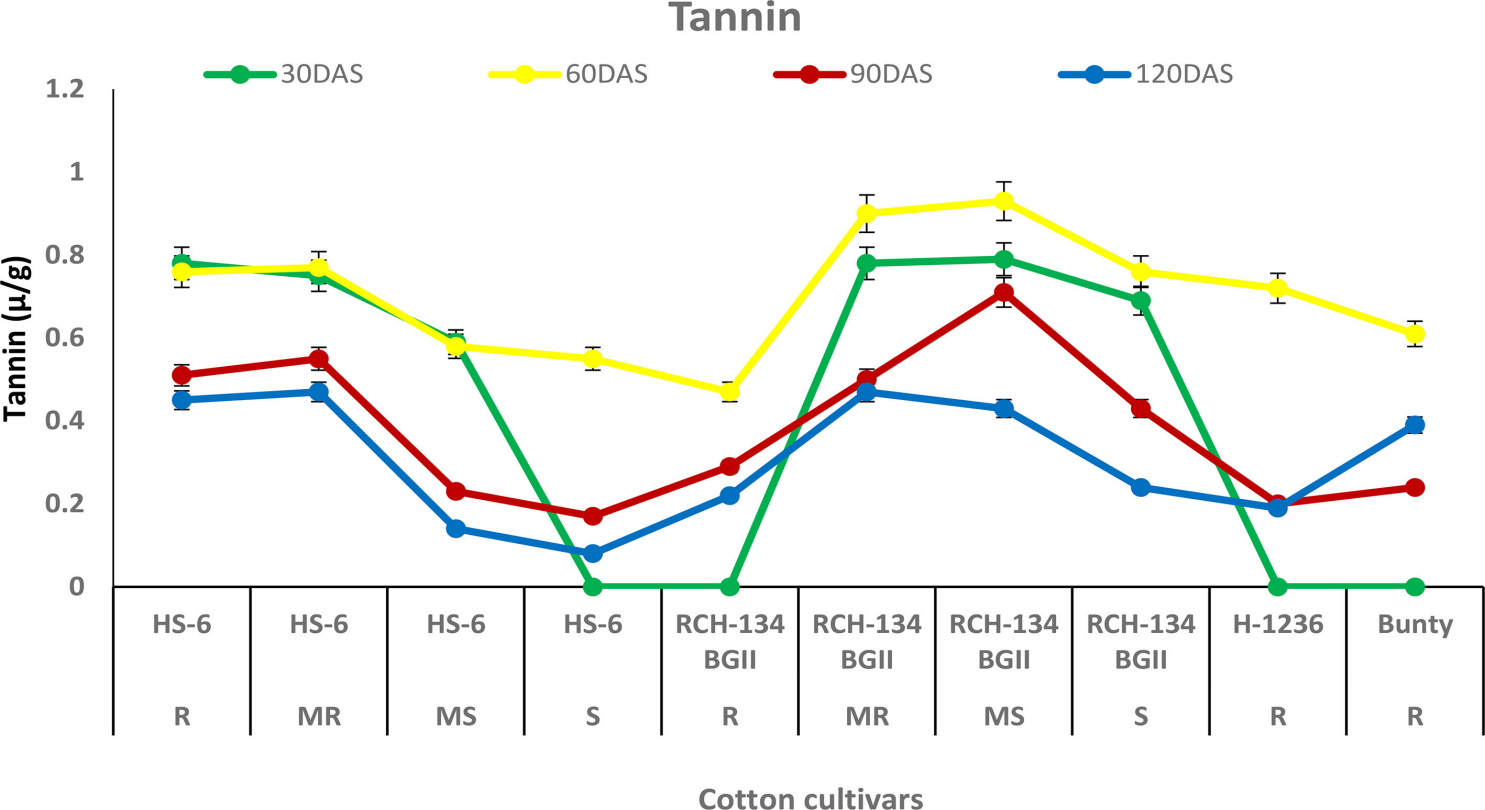
Tannin in resistant and susceptible cultivars of cotton estimated at 30, 60, 90, and 120 days after sowing. The susceptible cultivars were Non-Bt (HS 6) and Bt (RCH 134 BGII) with varied disease reactions. Non-Bt H1236 and Bt Bunty were taken as resistant cotton cultivars.

The tannin content increased in all cultivars up to 60 DAS, after which it decreased over time at 90 and 120 DAS. Higher tannin content was observed in plants with fewer disease symptoms than in highly diseased plants (**Figure 6**). Healthy leaves of the susceptible hybrid HS 6 expressed a high tannin content (0.76 µg/g) compared to highly diseased plants with grade 6 symptoms (0.47 µg/g) at 60 DAS. Similarly, symptomless leaves of the susceptible Bt cultivar RCH 134 BG-II exhibited 0.93 µg/g tannin compared to the highly diseased plants of RCH 134 BG-II, which had 0.61 µg/g tannin.

### 3.7 Disease progression

The disease progression on two cultivars was observed weekly. The data presented in **Table 2** show that disease progression reached a maximum of 38.4% up to 37 DAS in cultivar HS-6, and a maximum of 60.3% up to 44 DAS in RCH 134 BG-II in the first-year trial, after the appearance of the disease on 23 DAS. Thereafter, disease progression declined, and plants were fully infected after 58 days of sowing. Similarly, observations recorded in the second-year trials showed that disease progression reached a maximum of 33.0% on HS-6 and 34.4% on RCH 134 BG-II up to 51 DAS, after the appearance of disease on 30 DAS. Thereafter, not much disease progression was recorded, and plants were fully infected. Perusal of data from both the trials revealed that the disease initiated around the 4th to 5th week and reached its maximum within 9–10 weeks of crop age in both the cultivars. It indicates that there was no significant difference between hybrid and Bt cotton regarding CLCuD infection and multiplication.

## 4 Discussion

In this study, sugar was investigated as part of the biochemical response to the virus due to its role as a source of energy for various physiological activities. However, our findings indicate that the total sugar content in resistant and susceptible cotton plants did not have a significant role in resistance against the virus. Interestingly, we observed variations in sugar content in plants, regardless of their reaction to the disease. Previous studies have reported high sugar content in susceptible plants (Klement and Goodman, 1967; Jayapal and Mahadevan, 1968; Patil et al., 2010; Lal et al., 2021), while in some cases, no significant association between sugar and disease resistance has been observed (Ashfaq et al., 2014). The limited understanding of the function of sugar in disease resistance remains a hindrance. While our study did not find sugar to be a contributing factor to the biochemical response against CLCuV, it may be worth investigating its role in the host–pathogen interaction phenomenon.

Protein biosynthesis in host plant interaction usually occurs in the incompatible reaction. It has been suggested that the high protein content in the infected plant may be due to the activation of host defense mechanisms between the host and pathogen (Agrios, 2005). The present study found that resistant cultivars H 1236 and Bunty had a high protein content, and even the less diseased plants of susceptible cultivars showed an increase in protein compared to highly diseased plants. This observation clearly indicates that protein plays a role in resistance against CLCuV infection. Our results are in agreement with previous findings that support the association of protein as one of the defense responses against CLCuV in cotton plants (Beniwal et al., 2006; Acharya and Singh, 2008; Siddique et al., 2014).

The physiological responses of stressed plants differ, and chlorophyll is one of the components that may be involved in their response to disease (Kumar et al., 2022). The present study demonstrated that chlorophyll levels increased with disease index and plant age. Research has shown that chlorophyll levels increase in diseased plants due to their role in the intercellular movement of viruses through symplastic pathways within the plant (Zhao et al., 2016). However, our study’s findings did not align with this movement of the virus. Nevertheless, we observed a higher rise in chlorophyll in mature susceptible plants than in younger plants in all cultivars, indicating the accumulation of chlorophyll under CLCuV infection. Earlier studies also supported an increase in chlorophyll in susceptible plants (Devlin and Witham, 1983; Reddy et al., 2005; Kandhasamy et al., 2010; Ashfaq et al., 2014; Lal et al., 2021). Limited information exists on the role of chlorophyll in disease reaction; however, studies on its mechanism may provide insight into chlorophyll’s involvement in both diseased and mature plants.

In host–pathogen interactions, phenols are considered to be the most important components in the defense response and play a key role in imparting resistance to plant diseases (Kumar et al., 2021). Our study found that resistant cultivars had a higher phenol content compared to susceptible cultivars. These findings are consistent with previous studies that have demonstrated an increase in phenol levels in resistant cultivars of cotton against CLCuV, indicating the secondary level of defense line in the plant (Ajmal et al., 2011). The possible explanation for this is that phenolic compounds help in the synthesis of lignin and suberin, providing mechanical strength to host cells and acting as physical barriers against pathogens (Ngadze et al., 2012; Singh et al., 2014). In our study, the increase in phenolic content also suggests a resistant reaction against CLCuV. Similar results have been observed in okra with high phenol content in the resistant reaction against OYVMV (Manju et al., 2021). However, the classification of phenolics involved in resistance was beyond the scope of this study. Nevertheless, previous research has suggested that phenol content could be used as reliable biochemical markers for early selection of genotype resistant to OELCuD (Yadav et al., 2020).

Gossypol is a toxic terpenoid aldehyde (TA) compound that is released in cotton and is known for its insecticidal properties against insect pests (Heinstein et al., 1979; Widmaier et al., 1980). As CLCuV is transmitted through whiteflies, we investigated the gossypol levels in cotton cultivars in the present study. Surprisingly, we found no evidence of any change in gossypol content in resistant and susceptible cotton plants against CLCuV. Although gossypol is toxic to various insect-pests, nematodes, and fungi (Bell, 1986), there are opportunities to explore its potential antimicrobial and antiviral properties in cotton. Similarly, tannins are an important group of secondary metabolic compounds that play a significant role in plant defense mechanisms against diseases and insect pests (Swain, 1979). In our study, we also analyzed the tannin content and found that susceptible cultivars exhibited a decrease in tannin content with an increase in disease, while resistant cultivars showed a higher tannin content. These results are consistent with the previous findings that tannins play a crucial role in imparting resistance to cotton cultivars against CLCuV (Beniwal et al., 2006; Acharya and Singh, 2008).

The results showed that while sugar and gossypol levels did not provide clear information on the resistance or susceptibility of the plants, other compounds such as protein, chlorophyll, phenols, and tannins can be used as markers for resistance against CLCuV. These findings offer insight into the role of primary and secondary metabolites in hybrid and Bt cotton’s resistance to CLCuV.

## 5 Conclusions

The results showed that resistant cultivars activate proteins, phenols, and tannins against CLCuV, while sugar, gossypol, and chlorophyll, associated with basic physiological and metabolic mechanisms, did not play a significant role in resistance against CLCuV infection in cotton plants. Among the primary biochemical compounds, the activity of proteins was proposed as the first line of defense, while the secondary level of defense line in resistance exhibited the activity of phenols and tannins as the most significant in imparting resistance against CLCuV in cotton. The present study highlights the importance of biochemical studies in understanding the changes occurring in plants under biological stress due to viral infections. The findings provide valuable biochemical information to understand the mechanism of action involved in resistance against CLCuV and can serve as a source of information for the development and differentiation of resistance in cotton plants against CLCuV.

## Data availability statement

The original contributions presented in the study are included in the article/**Supplementary Material**. Further inquiries can be directed to the corresponding authors.

## Author contributions

PC and NM: Conceptualization, methodology, investigation, and writing—original draft preparation. RC and AK: Methodology, software, and visualization. RKT and RK: Reviewing and editing. ML: Data curation and revision. HS: Validation. All authors contributed to the article and approved the submitted version.

## Supplementary material

Supplementary Figure 1 CLCuD symptoms on infected plant **(A)** and enation at abaxial side of leaf with white fly infestation **(B)**.
