# Supplementary figures and images for: Utilization of primary and secondary biochemical compounds in cotton as diagnostic markers for measuring resistance to cotton leaf curl virus
Source: Front Plant Sci. 2023 Jun 6;14:1185337. doi: 10.3389/fpls.2023.1185337 (PMC10280379; doi:10.3389/fpls.2023.1185337)

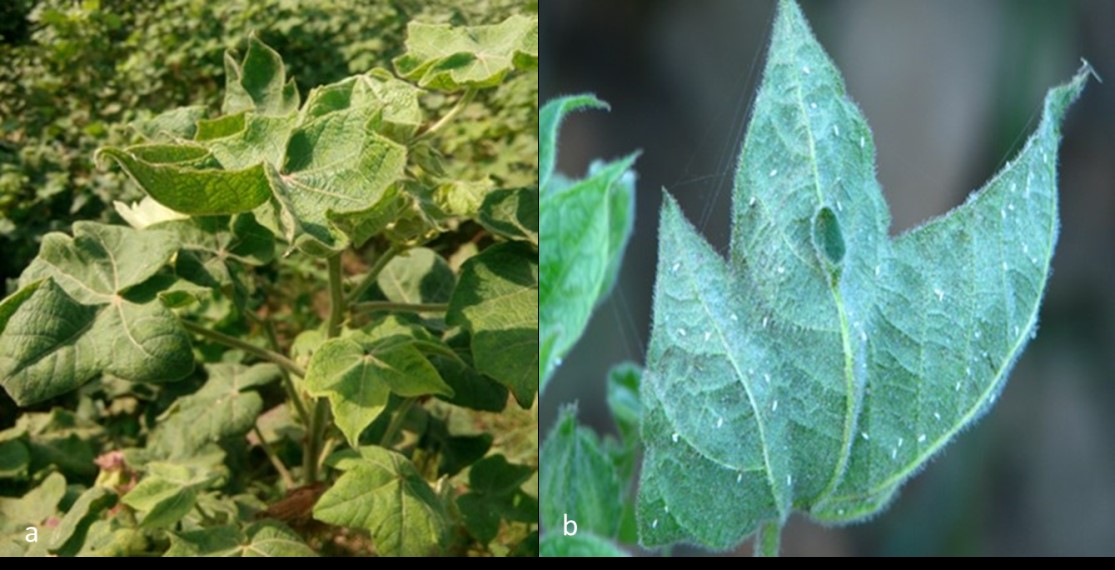

Supplement: Supplementary Figure 1 — CLCuD symptoms on infected plant (A) and enation at abaxial side of leaf with white fly infestation (B). [file Image_1.jpg]
